# Self-Concept With Cross-Cultural Perspective: 36–72-Month-Old Preschool Children in Turkey and Germany

**DOI:** 10.3389/fpsyg.2022.821074

**Published:** 2022-05-23

**Authors:** Busra Celikel, Aysel E. Çoban

**Affiliations:** Department of Early Childhood Education, Institute of Educational Sciences, Hacettepe University, Ankara, Turkey

**Keywords:** self-concept, preschool children, early childhood, Turkey – Germany, cross-cultural

## Abstract

Children attending preschool education in Turkey and Germany have different cultural environments and education systems. This study aimed at investigating the self-concept of 36–72- month-old preschool children in Turkey, a country with a collectivist culture, and Germany, a country with an individualistic culture. Participants were 433 children (234 female, 199 male) from Turkey and 206 children (102 female, 109 male) from Germany. Three dimensions of self-concept were measured: ability-based, social, and physical. The Self-Concept Questionnaire for Children (Preschool Version) was used for data collection. This process lasted about 5 months. Country (Turkey vs. Germany) and sex (male vs. female) of the child were analyzed as independent variables, and the three dimensions of self-concept (i.e., ability-based, social, and physical) were analyzed as dependent variables. As normality assumption was not met for the subgroups, the Mann–Whitney *U* test was applied for statistical analysis. Results showed significant differences between children from Turkey and children from Germany in two self-concept dimensions (i.e., ability-based and physical). It was determined that there were no differences between the two countries in social self-concept. It was also determined that there were differences in the ability-based self-concept dimension in 36–72-month-old children depending on gender in Turkey, in favor of male children. On the other hand, it was determined that there were no differences depending on gender in any of the self-concept dimensions in Germany. It was concluded that culture was a factor leading to differentiation in some dimensions of preschool children’s self-concept.

## Introduction

The most important factor in the formation of personality, and therefore behaviors, is the self (Lecky, 1961; Erikson, 1968; Gizir and Baran, 2003; Cevher and Bulus, 2006). Self is an individual’s awareness and perception of his/her own personality (Morgan, 1961; Lindgren, 1969; Aslan, 1992; Balkis Baymur, 2014). The descriptive perception of the self is the self-concept, and the evaluative perception is self-esteem. Even if there are slight differences between self-concept and self-esteem, it is difficult to distinguish between the meanings of these concepts (Brinthaupt and Erwin, 1992; Bracken and Lamprecht, 2003). The empirical evidence provided by many studies suggest that they are highly related and hardly separable constructs, which is probably why many researchers tend to use the terms interchangeably (Shavelson and Bolus, 1982; Brinthaupt and Erwin, 1992; Bracken and Lamprecht, 2003). Self-concept is any kind of description of the self made verbally, through paintings, or using different methods (Yamamoto, 1972; Epstein, 1973; Oyserman et al., 2012). The first scientific theory of self-concept argues that the self, in its broadest sense, is all that an individual can call “I” or “me” (James, 1952). Self-concept is a structure acquired over time and is shaped by experiences. Early childhood is the most critical time for the formation and shaping of self-concept (Purkey, 1970). The first step of self-concept formation is the infant’s simple distinction of self by first distinguishing between “me” and “non-me” (Malpass et al., 1965). John Bowlby’s attachment theory reports that the infant’s self-awareness begins to develop through interactions between the infant and the main caregiver, as well as the caregiver’s support for the infant. For instance, if the caregiver provides a secure attachment to the baby, he/she develops a positive self-concept of being valuable, whereas a negative self-concept of being worthless is created if the caregiver ignores the baby (Vasta et al., 2004). A clearer distinction of a child’s own self is achieved by the onset of language development and by the emergence of the child’s ability to describe the objects in the environment, his/her own body, and own existence. The child’s self-awareness increases with the development of perceptual skills and language, which happens around the age of two (Malpass et al., 1965). Self-concept definitions begin with simple descriptions of the self from the age of three. Three-year-old children can create a portrait of themselves by making concrete mental descriptions of the self (Harter, 1999). It has been determined that young children aged between 48 and 66 months can define self-concepts. Moreover, it is possible to distinguish between multiple dimensions of self-concept in children (Marsh et al., 2002). Children begin to recognize and define their own selves at an early age. Therefore, it is necessary to support the development of positive self-concepts from the moment children realize themselves because children develop a positive or negative self-concept in accordance with the feedback and definitions they receive from their environment. Children who develop a negative self-concept isolate themselves from life with a sense of worthlessness, while children who develop a positive self-concept become individuals who are more active and psychologically more resilient (McConnell et al., 2009). In addition, the self-concept is also in mutual interaction with academic achievement (Wolff et al., 2018; Sewasew and Schroeders, 2019). Children with positive self-concept show better academic achievement than children with negative self-concept (Tang, 2011; Susperreguy et al., 2017). When children have a positive self-concept of a subject, they take a more focused approach to that subject and this relates to achievement (Cai et al., 2018). Having a positive self-concept allows children to be aware of their unique characteristics, establish positive social relationships with people around them, and evaluate events correctly. On the other hand, having a negative self-concept negatively affects the development of body and sexual identity (Aydogdu et al., 2021). In addition to these, the ability-based self-concept in math in childhood influences the future professional preferences of children (Heyder et al., 2019). It is crucial to support positive self-concept in early childhood so that children can become individuals with positive characteristics in societies and make correct decisions when they become adults (Yusop et al., 2018). As a result, it can be said that self-concept is a structure that affects the whole life.

There is an important question in the literature, whether the self is a unidimensional or a multidimensional construct (Marsh and Shavelson, 1985; Chen et al., 2020). While some models are based on the conceptualization of the self as a global evaluative component (Baumeister et al., 2003), others follow a multidimensional approach with different but related dimensions (Shavelson et al., 1976; Garcia et al., 2018; Chen et al., 2020). Although the self appears as a whole, it consists of different dimensions. It is accepted that it is useful to investigate the dimensions developmentally constituting the self separately (Schaffer, 2004). There are different self-concept dimensions such as social, affect, family, academic, physical, and competence that constitute the self-concept. These dimensions need to be taken into account to properly address the self-concept (Bracken and Lamprecht, 2003). Different theoretical dimensions like academic, social, emotional, family, and physical have been identified following a multidimensional approach (Garcia et al., 2018; Fuentes et al., 2020). The multidimensional conceptualization of self-concept has been confirmed in different continents such as Europa (Murgui et al., 2012), Latin America (Garcia et al., 2011, 2018), United States (Garcia et al., 2013), and China (Chen et al., 2020). The multidimensional approach to the self may offer more specific predictions of psychosocial functioning than the global approach (Marsh and O’Mara, 2008; Garcia et al., 2018; Chen et al., 2020). For example, studies show that physical self-concept is related to sports practice (Murgui et al., 2012), academic self-concept is related to academic achievement (Marsh and O’Mara, 2008), social self-concept is negatively related to drug use (Fuentes et al., 2020), and the family dimension of self is positively related to parental warmth (Martinez et al., 2021). In this regard, theoretically consistent relationships between parental practices and children’s psychosocial development have been described (Fuentes et al., 2015; Gallarin et al., 2021). One of the main responsibilities of parents is raising children (Sandoval et al., 2022). The family dimension of the self can explain the impact of parents (e.g., indulgent, authoritative, authoritarian, and neglectful) on children’s development (Martinez et al., 2021). Shavelson et al. (1976) divide the self-concept into two general categories: academic self-concept and non-academic self-concept. Academic self-concept, which is also referred to as ability-based self-concept, covers language, history, mathematics, and science, while non-academic self-concept includes social, emotional, and physical self-concept. Social self-concept is formed by the interaction of children with their peers and the people they care about. Emotional self-concept is formed through special emotional situations. On the other hand, physical self-concept consists of physical competence and views on appearance. Although preschool children can distinguish and interpret academic self-concept, social self-concept, and physical self-concept, they cannot distinguish emotional self-concept (Engel, 2015). Therefore, this study investigated the ability-based, social, and physical self-concept of children, and excluded the emotional self-concept.

Self-concept, which consists of various dimensions, can be affected by the culture of the society to which the child belongs (Shavelson et al., 1976; Robins et al., 1998; Smith and Hart, 2010; European Economic and Social Committee, 2016; Ahmed and Zaman, 2019; Vaughn, 2019). Every society has its own cultural values. However, societies are generally divided into two groups: individualist and collectivist in the context of culture (Hofstede, 2001; Kashima et al., 2001; Oyserman et al., 2002; Triandis, 2007; Kagitcibasi, 2012). While Turkey is one of the countries considered to have a collectivist culture, Germany is one of the countries considered to have an individualistic culture, and self-concept of people differs according to whether they come from an individualistic or collectivist culture (Hofstede, 2001; Kagitcibasi, 2012). However, even though societies are classified according to culture, it is possible for a society to contain both individualistic and collectivist cultures simultaneously (Oyserman et al., 2002).

The basis of the collectivist culture is to be a member of a group, obey the social rules, and care about the welfare of the society. Individualistic culture, on the other hand, adopts the view that the wishes of the individuals themselves are significant and that society exists for the welfare of the individual (Erez, 1993; Oyserman et al., 2002; Oyserman and Lee, 2008). In terms of self-concept in collectivist and individualistic cultures, the relational and collectivist self is generally observed in collectivist culture, while the individualistic self is seen more often in individualistic culture (Kagitcibasi, 2010; Kagitcibasi and Cemalcilar, 2014). The relational self is based on establishing strong ties in relationships and prioritizing the views of individuals establishing relationships. The collectivistic self is related to people’s adaptation to the groups they belong to and behaving according to group expectations. The individualistic self, on the other hand, considers concepts of individual goals, unique personal characteristics and abilities, and self-confidence important (Sedikides and Brewer, 2001; Kagitcibasi, 2010; Chen et al., 2011; Sedikides et al., 2011, 2013; Nehrlich et al., 2018). This study focuses on preschool children’s self-concept in a cross-cultural perspective of collectivist and individualistic culture and highlights the culture-influenced dimensions of the self-concept.

### The Present Study

Children attending preschool education in Turkey and Germany have different cultural environments and education systems. The comparison of the self-concepts of these children is important in terms of discussing their differences and similarities in the self-concept levels by bringing a cross-cultural perspective to self-concept in children. Determining whether children’s self-concepts are at a low level (or at risk of being low) or at a normal level will facilitate the early taking of necessary precautions and supporting the positive development of self-concept. Addressing the self-concept with a cross-cultural comparative approach will be the basis for more diverse results and interpretations than investigating it within a single culture (Kagitcibasi, 2012). Nowadays, along with globalization, societies are interacting with each other more and more. Moreover, children from different cultures can also receive education in kindergartens in various countries. Studying the self-concept based on culture will contribute to the consideration of cultural differences by stakeholders and policymakers in the preschool education system. Furthermore, in this study, children’s self-concepts are also investigated cross-culturally depending on sex. Cross-cultural research of gender differences allows deciphering gender stereotypes and explains why certain behaviors of males and females differ across certain cultures in early childhood (Wong and Vanderlaan, 2020). In this regard, this study aims at determining the self-concept levels of 36–72-month-old children attending preschool education in Turkey and Germany and determining whether there is a significant difference between their self-concepts. To this end, the research questions and hypotheses of the present study are as follows:

1. Is there a significant difference between the scores obtained by children in Turkey and Germany from the ability-based, social, and physical self-concept dimensions? It was expected that children in individualist German culture would be at a higher level than Turkish children in collectivist culture in all the self-concept dimensions.

2. Do the levels of children in Turkey and Germany differ significantly by sex according to the scores they take in the ability-based, social, and physical self-concept dimensions? It was expected that males’ ability-based and physical self-concepts would be higher than females in both countries, and the social self-concept would be the same for females and males.

## Materials and Methods

Two independent variables, country (Turkey vs. Germany) and sex (male vs. female) of the child were analyzed to determine whether the self-concept of 36–72-month-old children (i.e., ability-based, social, and physical dimensions) attending preschool education in Turkey and Germany varied depending on culture and sex. Considering Turkey, a country with a collectivist culture, and Germany, a country with an individualistic culture (Chiu, 1992; Garcia et al., 2019; Chen et al., 2020).

### Participants

An *a priori* power analysis (Faul et al., 2009) was estimated that a minimum of 448 participants was needed to conduct the study with a medium-small effect-size with a high power (α = 0.05, 1–β = 0.95; *d* = 0.35) in a univariate *t*-test with two groups (Garcia Perez et al., 1999; Garcia et al., 2008; Veiga et al., 2021). Present study sampling the self-concept of 36–72-month-old children attending preschool education in public and private preschools in one large city with a total of 54,346 preschool children in Turkey and in one medium-sized city with 10,775 preschool children in Germany. The ending study sample consisted of 639 preschool children aged 36–72 months in public and private preschools in Turkey and Germany. A total of 433 (68%) children (*M* = 62.39-month-old, 54% female, 46% male) from Turkey and a total of 206 (32%) children (*M* = 57.2-month-old, 49.5% female, 50.5% male) from Germany. As the first sample selection criterion, children were selected in such a way as to have a similar number of males and females in both countries. As the second sample selection criterion, it was ensured that the participant sample included children from different parts of the city who received education in different kinds of educational institutions to reflect the entire socio-cultural environment of the city children lived in. With the present study sample of 639 children, a sensitivity power analysis (suitable for Mann–Whitney *U* test) among the 433 children from Turkey and 206 children from Germany guaranteed the detection of a medium-small effect size of 0.310 (*d* = 0.310, α = 0.05, 1–β = 0.95) (Faul et al., 2009).

### Procedure

The data collection process was carried out in two stages, one in Germany and one in Turkey. Required ethical permissions were obtained before starting the data collection process. Voluntary participation forms were signed by the families of the children. Only the children whose parents allowed them to participate and themselves volunteered to participate in the study were included. For the data collection in Germany, the original questionnaire was administered to the children by a native German-speaking scientist. Before starting the data collection process in Turkey, one-on-one training was received from a science expert in the field of preschool education, who applied the scale in Germany. After the adaptation of the questionnaire to Turkish, the exact procedure in Germany was followed in Turkey: a researcher sat in a room alone with the child and applied the questionnaire. Before the application, a short pre-study was performed with each child to determine the volunteering and enthusiastic children to include in the main study.

### Measures

The Turkish version of the Self-Concept Questionnaire for Children (Preschool Version), originally named Selbst Konzept Fragebogen Fur Kinder (Version Kita) (Engel, 2015), was used in the part of the study conducted in Turkey. In the part of the study conducted in Germany, the original scale was used. Both the original version and the Turkish version of the scale consisted of three dimensions: ability-based self-concept (e.g., “Do you like learning new things? How much do you like it?”), social self-concept (e.g., “Do you like being with other children? How much do you like it?”), and physical self-concept (e.g., “Do you get tired often? How often?”). The ability-based self-concept dimension consisted of 11 items, the social self-concept dimension consisted of seven items, and the physical self-concept dimension consisted of eight items. The original and adapted scale were Likert-type and consisted of a total of 26 items. Each item in the scale was scored as follows: very little = 1/a little = 2/quite = 3/a lot = 4. According to the scores obtained from the dimensions, the participants were defined as having low self-concept, needing support for self-concept, and normal self-concept. Both the original and adapted scales were valid and reliable. In the original scale, the inter-reliability co-efficient was found to be 0.79 for ability-based self-concept, 0.74 for social self-concept, and 0.72 for physical self-concept for German children (Engel, 2015, p. 143). In the adapted scale, the inter-reliability co-efficient was found to be 0.68 for ability-based self-concept, 0.56 for social self-concept, and 0.64 for physical self-concept for Turkish children. In the original scale, the test-retest correlation coefficient was found to be 0.56 for ability-based self-concept, 0.53 for social self-concept, and 0.34 for physical self-concept for German children (Engel, 2015, p. 143). In the adapted scale, the test-retest correlation coefficient was found to be 0.89 for ability-based self-concept, 0.82 for social self-concept, and 0.76 for physical self-concept for Turkish children. In addition to these, the item analysis was performed on the scale adapted to Turkish. Total item correlation values were found to be ranging from 0.41 to 0.54 for ability-based self-concept, from 0.28 to 0.62 for social self-concept, and from 0.43 to 0.65 for physical self-concept. In the interpretation of the total item correlation value, it is accepted that items with a correlation coefficient of 0.30 and above are sufficient to distinguish the characteristic to be measured (Buyukozturk, 2010; Erkus, 2012). In the current study, it was determined that only one item was below 0.30. However, considering all of the scale items, this item was not removed because it should be found theoretically and it was found appropriate to keep it in line with the expert opinions. In the analysis of the lower and upper groups, the fact that the *t*-values of the differences between the groups are significant is evidence of the discriminatory power of the item (Erkus, 2012). As a result, when the findings obtained from the item analysis were evaluated according to the criteria related to the total correlation and the 27% lower-upper group comparisons, it was determined that all of the items included in the dimensions of the self-concept scale were distinctive.

### Data Analysis

Before conducting the data analysis, the necessary assumptions regarding missing data, outliers and normality were tested and reported. Missing data analysis was performed for the missing data in the data set. First, the amount of missing data was determined. It was seen that the amount of missing data for all items in the scale did not exceed 5% of the total data both in Turkey and Germany. Little’s MCAR test was performed to determine whether there was a pattern in the missing data or not. As a result of the test, the null hypothesis was accepted, and it was determined that the missing data were randomly distributed (*p* > 0.05). The analysis was continued by assigning data to replace the missing data with the multiple imputation method. The z-scores were used to analyze the outliers. It was determined that the z scores calculated for each dimension were within the range of ±4 points.

In terms of normal distribution, the Kolmogorov–Smirnov test results, skewness coefficient and standard error values and histograms were analyzed. For the measures used in this study, the Kolmogorov–Smirnov test was performed on both German data and Turkish data as the sample size was bigger than 50.

The sample size was larger than 50 for the measures used in the collection of both the Turkish data and German data in this study. Therefore, the Kolmogorov–Smirnov test was performed. In addition to the normality test, information about the skewness coefficient and the standard error was presented in Table 1.

**TABLE 1:** Results on the normal distribution for Turkey and Germany data.

		Kolmogorov–Smirnov			Skewness		
		Statistic	df	Significance	Statistic	Std. error	Statistic/Std. error
Turkey	Ability-based self-concept	0.136	433	0.000	–1.103	0.117	9.43
	Social self-concept	0.115	433	0.000	–0.652	0.117	5.57
	Physical self-concept	0.107	433	0.000	–0.602	0.117	5.15
Germany	Ability-based self-concept	0.144	206	0.000	–0.892	0.169	–5.28
	Social self-concept	0.142	206	0.000	–0.780	0.169	–4.62
	Physical self-concept	0.079	206	0.003	0.361	0.169	2.13

It was observed that the scores obtained from the dimensions of the scale were not normally distributed in either Turkey or Germany data. Mann–Whitney *U* test was used to compare the two groups.

In addition to the scores obtained from the entire group, the distribution of the data for the subgroups to be compared was also investigated. The Kolmogorov–Smirnov test and information on skewness values regarding the results obtained from the analysis of the gender subgroups, including female and male children, were presented in Table 2.

**TABLE 2:** Results on the normal distribution for subgroups.

			Kolmogorov–Smirnov		Skewness	
			Statistic	Significance	Statistic	Std. error
Female children	Turkey	Ability-based self-concept	0.139	0.000	–0.803	0.159
		Social self-concept	0.106	0.000	–0.598	0.159
		Physical self-concept	0.102	0.000	–0.621	0.159
	Germany	Ability-based self-concept	0.155	0.000	–0.723	0.239
		Social self-concept	0.174	0.000	–0.785	0.239
		Physical self-concept	0.073	0.200	0.436	0.239
Male children	Turkey	Ability-based self-concept	0.158	0.000	–1.533	0.172
		Social self-concept	0.126	0.000	–0.699	0.172
		Physical self-concept	0.115	0.000	–0.583	0.172
	Germany	Ability-based self-concept	0.139	0.000	–0.927	0.237
		Social self-concept	0.126	0.000	–0.785	0.237
		Physical self-concept	0.109	0.004	–0.815	0.237

In the analysis of the subgroups, it was determined that the data were not normally distributed among the subgroups. For this reason, the Mann–Whitney *U* test was used. Since *Z* values should be reported when the number of observations at the levels of independent variables is above 20 in the Mann–Whitney *U* test, *Z* values were reported in the current study (Buyukozturk et al., 2015).

## Results

The results obtained in the dimensions of ability-based self-concept, social self-concept, and physical self-concept by 36–72-month-old children in Turkey and Germany attending preschool education were presented in Table 3.

**TABLE 3:** Frequency and percentage distribution of children in Turkey and Germany from the dimensions of the self-concept questionnaire for children.

Dimensions	Country	Low		Risk group		Normal		χ^2^	*p*
		*f*	%	*f*	%	*f*	%		
Ability-based self-concept	Turkey	12	2.8%	44	10.2%	377	87.1%	10.331	0.006*
	Germany	17	8.3%	24	11.6%	165	80.1%		
Social self-concept	Turkey	14	3.2%	52	12.0%	367	84.8%	3.140	0.208
	Germany	12	5.8%	29	14.1%	165	80.1%		
Physical self-concept	Turkey	10	2.3%	51	11.8%	372	85.9%	149.214	0.000**
	Germany	48	23.3%	74	35.9%	84	40.8%		

*f, frequency; χ^2^, Chi-squared statistic; p, significance.*

*p < 0.05; **p < 0.001.

Table 3 showed that in the ability-based self-concept dimension, 2.8% (12 children) of the children in Turkey were in the low group, 10.2% (44 children) were in the risk group, and 87.1% (377 children) were included in the group defined as normal. On the other hand, 8.3% (17 children) of the children in Germany were in the low group, 11.6% (24 children) were in the risk group and 80.1% (165 children) were in the normal group. In terms of the ability-based self-concept dimension, there were significant differences (*p* < 0.05) between the Turkish and German children in the low, risk, and normal groups. It was considered that this was due to the fact that the low group in Turkey differed significantly from the low group in Germany.

In the social self-concept dimension, 3.2% (14 children) of the children in Turkey were in the low group, 12% (52 children) were in the risk group, and 84.8% (367 children) were in the normal group. On the other hand, 5.8% (12 children) of children in Germany were in the low group, 14.1% (29 children) were in the risk group and 80.1% (165 children) were in the normal group. There was no significant difference in the social self-concept dimension.

In the physical self-concept dimension, it was determined that 2.3% (10 children) of children in Turkey were in the low group, 11.8% (51 children) were in the risk group and 85.9% (372 children) were in the normal group. On the other hand, 23.3% (48 children) of children in Germany were in the low group, 35.9% (74 children) were in the risk group and 40.8% (84 children) were in the normal group. The results of the chi-square test revealed that there were significant differences between the Turkish and German children in the low, risk, and normal groups in terms of the ability-based self-concept dimension (*p* < 0.001). It was considered that this might be because there were more children in Germany in the low and risk groups, and more children in Turkey in the normal group.

Mann–Whitney *U* test was conducted to determine whether there was a significant difference between children according to countries and the results were presented in Table 4.

**TABLE 4:** Mann–Whitney *U* test results regarding children’s scores from the dimensions of the self-concept questionnaire for children by country.

	Country	*n*	Mean rank	Sum of ranks	*Z*	*p*
Ability-based self concept	Germany	206	296.85	61151.00	2.196	0.028*
	Turkey	433	331.01	143329.00		
Social self-concept	Germany	206	318.93	65699.00	0.102	0.919
	Turkey	433	320.51	138781.00		
Physical self-concept	Germany	206	183.83	37869.50	12.878	0.000**
	Turkey	433	384.78	166610.50		

*p < 0.05, **p < 0.001.

As can be seen in Table 4, the scores of 36–72 month-old preschool children in the ability-based self-concept dimension differed significantly depending on the country (*Z*_*AbilSC*_ =  2.196;*p* =  0.028). This significant difference was in favor of the preschool children in Turkey for this dimension (Mean Rank = 331.01). Similarly, children’s scores in the physical self-concept dimension differed significantly depending on the country (*Z*_*PhySC*_ =  12.878;*p* =  0.001). This significant difference was in favor of the preschool children in Turkey for this dimension (Mean Rank = 348.78). There was no significant difference between countries in terms of the scores taken from the social self-concept dimension (*Z*_*SocSC*_ =  0.102;*p* > 0.05).

In addition to the analysis of dimensions of children’s self-concept according to two different cultures, children’s levels of self-concept were analyzed according to gender to see whether there were differences in the self-concept dimensions of the children from different cultures. Frequency and percentage distributions in the dimensions of the self-concept questionnaire for children by gender were presented in Table 5.

**TABLE 5:** Frequency and percentage distribution in the dimensions of the self-concept questionnaire for children by gender.

	Dimensions	Gender	Low		Risk group		Normal		χ^2^	*p*
			*f*	%	*f*	%	*f*	%		
Turkey	Ability-based self-concept	Female	6	2.6%	31	13.2%	197	84.2%	5.336	0.069
		Male	6	3.0%	13	6.5%	180	90.5%		
	Social self-concept	Female	6	2.6%	28	12.0%	200	85.4%	0.736	0.692
		Male	8	4.0%	24	12.1%	167	83.9%		
	Physical self-concept	Female	3	1.3%	30	12.8%	201	85.9%	2.797	0.247
		Male	7	3.5%	21	10.6%	171	85.9%		
Germany	Ability-based self-concept	Female	13	12.7%	14	13.7%	75	73.5%	6.776	0.034*
		Male	4	3.8%	10	9.6%	90	86.5%		
	Social self-concept	Female	7	6.9%	17	16.7%	78	76.5%	1.667	0.435
		Male	5	4.8%	12	11.5%	87	83.7%		
	Physical self-concept	Female	20	19.6%	39	38.2%	43	42.2%	1.578	0.454
		Male	28	26.9%	35	33.7%	41	39.4%		

*f, frequency; χ^2^, Chi-squared statistic; p, significance.*

*p < 0.05.

Table 5 showed that for Turkey in the ability-based self-concept dimension, 2.6% (6 children) of the females were in the low group, 13.2% (31 children) were in the risk group, and 84% (197 children) were in the normal group. On the other hand, 3% (6 children) of the males were in the low group, 6.5% (13 children) were in the risk group, and 90.5% (180 children) were in the normal group. In the social self-concept dimension, 2.6% (6 children) of the females were in the low group, 12% (28 children) were in the risk group, and 85.4% (200 children) were in the normal group. On the other hand, 4% (8 children) of the males were in the low group, 12.1% (24 children) were in the risk group, and 83.9% (167 children) were in the normal group. For the physical self-concept dimension, 1.3% of the females (3 children) were in the low group, 12.8% (30 children) were in the risk group, and 85.9% (201 children) were in the normal group. On the other hand, 3.5% (7 children) of the males were in the low group, 10.6% (21 children) were in the risk group, and 85.9% (171 children) were in the normal group. There were no significant differences between the female and male children in terms of their placement in the low, risk, and normal groups of the self-concept dimensions for Turkey.

For Germany, in the ability-based self-concept dimension, 12.7% (13 children) of the females were in the low group, 13.7% (14 children) were in the risk group, and 73.5% (75 children) were in the normal group. On the other hand, 3.8% (4 children) of the males were in the low group, 9.6% (10 children) were in the risk group, and 86.5% (90 children) were in the normal group. For the social self-concept dimension, 6.9% (7 children) of the females were in the low group, 16.7% (17 children) were in the risk group, and 76.5% (78 children) were in the normal group. On the other hand, 4.8% (5 children) of the males were in the low group, 11.5% (12 children) were in the risk group and 83.7% (87 children) were in the normal group. In the physical self-concept dimension, 19.6% (20 children) of the females were in the low group, 38.2% (39 children) were in the risk group, and 42.2% (43 children) were in the normal group. On the other hand, 26.9% (28 children) of the males were in the low group, 33.7% (35 children) were in the risk group, and 39.4% (41 children) were in the normal group. There were no significant differences between the female and male children in terms of their placement in the low, risk, and normal groups of social and physical self-concept dimensions for Germany. However, there was a significant difference in terms of their placement in the low group (*p* < 0.05) of the ability-based self-concept dimension.

Mann–Whitney *U* test results regarding whether there was a significant gender difference between children in Turkey and Germany were presented in Table 6.

**TABLE 6:** Mann–Whitney *U* test results of children’s scores in the dimensions of the self-concept questionnaire for children by gender.

		Gender	*n*	Mean rank	Sum of ranks	*Z*	*p*
Turkey	Ability-based self-concept	Female	234	200.16	46838.00	3.050	0.002*
		Male	199	236.80	47123.00		
	Social self-concept	Female	234	219.19	51290.50	0.397	0.692
		Male	199	214.42	42670.50		
	Physical self-concept	Female	234	218.90	51222.00	0.343	0.732
		Male	199	214.77	42739.00		
Germany	Ability-based self-concept	Female	102	95.62	9753.50	1.885	0.059
		Male	104	111.23	11567.50		
	Social self-concept	Female	102	96.35	9827.50	1.713	0.087
		Male	104	110.51	11493.50		
	Physical self-concept	Female	102	104.95	10704.50	0.346	0.730
		Male	104	102.08	10616.50		

*p < 0.05.

As seen in Table 6, the scores of 36–72 month-old males in preschool education in Turkey in the dimension of ability-based self-concept differed significantly depending on gender (*Z*_*PhySC*_ = 3.050;*p* < 0.05). This significant difference was in favor of the males attending preschool (Mean Rank = 236.80). There was no significant difference by gender in the social self-concept and physical self-concept dimensions (*Z*_*SocSC*_ = 0.397;*p* > 0.05;*Z*_*PhySC*_ = 0.343;*p* > 0.05).

There was no significant difference between the scores taken from the dimensions of ability-based self-concept, social self-concept, and physical self-concept by the male and female children attending preschool education in Germany (*Z*_*PhySC*_ = 1.885;*p* > 0.05;*Z*_*SocSC*_ = 1.713;*p* > 0.05;*Z*_*PhySC*_=0.346;*p* > 0.05).

Mann–Whitney *U* test was conducted to determine whether there was a significant difference between the female and male children depending on the country and the results were presented in Table 7.

**TABLE 7:** Mann–Whitney *U* test results of the female and male children’s scores in the dimensions of the self-concept questionnaire by country.

		Country	*n*	Mean rank	Sum of ranks	*Z*	*p*
Female children	Ability-based self-concept	Turkey	234	174.38	40804.00	1.685	0.092
		Germany	102	155.02	15812.00		
	Social self-concept	Turkey	234	172.88	40453.50	1.256	0.209
		Germany	102	158.46	16162.50		
	Physical self-concept	Turkey	234	200.69	46961.50	9.213	0.000*
		Germany	102	94.65	9654.50		
Male children	Ability-based self-concept	Turkey	199	158.45	31531.50	1.782	0.075
		Germany	104	139.66	14524.50		
	Social self-concept	Turkey	199	148.17	29485.50	1.059	0.290
		Germany	104	159.33	16570.50		
	Physical self-concept	Turkey	199	184.56	36728.00	8.963	0.000*
		Germany	104	89.69	9328.00		

*p < 0.001.

When the values in Table 7 were analyzed, it was determined that the scores obtained from the physical self-concept dimension by the 36–72-month-old female children differed significantly by country (*Z*_*AbilSC*_=9.213,*p* = 0.001). This significant difference was in favor of the female children (mean rank = 200.69) attending preschool education in Turkey. There were no significant differences in the ability-based self-concept and social self-concept dimensions for the female children (*Z*_*PhySC*_ = 1.685;*p* > 0.05;*Z*_*SocSC*_=1.256;*p* > 0.05) in terms of country.

The scores obtained from the physical self-concept dimension of the male children differed significantly by country (*Z*_*PhySC*_ = 8.963;*p* = 0.001). This significant difference was in favor of the male children (mean rank = 184.56) attending preschool education in Turkey. There were no significant differences in the ability-based self-concept and social self-concept dimensions (*Z*_*AbilSC*_ = 1.782;*p* > 0.05;*Z*_*SocSC*_ = 1.059;*p* > 0.05) in terms of country.

## Discussion

While the expectations of society are important regarding the self-concept in collectivist cultures, the self-concept within individualistic cultures prioritizes the interests and desires of the individual. In a collectivist culture, thoughts about the self depend more on the expectations of others than the individual’s expectations (Oyserman et al., 2002; Oyserman and Lee, 2008; Erez, 2011). This suggests that people in a collectivist culture may be more judgmental of themselves and their desires/interests when they don’t coincide with society’s expectations. Some cross-cultural studies on the selves of individuals from countries with collectivist and individualistic cultures revealed that there were more positive approaches to the self in countries with an individualistic culture (Chiu, 1992; Schmitt and Allik, 2005). Accordingly, the first hypothesis of the present study was that the children in individualistic German culture would have higher scores in all self-concept dimensions than Turkish children, who were raised in a collectivist culture. However, the results showed that this hypothesis should be rejected. Yet, generally, there are similar structures of self in various cultures, not only a “relational and collectivist self” concept exists in collectivist cultures, nor merely an individualistic self exists in individualistic cultures. While there are general values that reflect every culture, there are also different self-structures in sub-groups of societies. For this reason, it is possible for values reflecting an individualistic and collectivist culture to coexist in some smaller social groups (Kagitcibasi, 2010; Kagitcibasi and Cemalcilar, 2014). Thus, it is perfectly possible to find the individualistic self-concept within collectivist cultures and the relational/collectivist self in individualistic cultures (Markus and Kitayama, 1991). In fact, individuals possess all of the individualistic, relational, and collectivist self-structures together in a hierarchy within themselves (Sedikides et al., 2013; Nehrlich et al., 2018). Every child goes through self-conflict processes required by their age at certain periods, despite the changing child-rearing styles depending on cultural values during the periods of self-development (Erikson, 1968). Therefore, it is more difficult to classify children’s self-constructs as individualistic, relational, or collectivist in comparison to adults. However, this study aimed at contributing to the literature by investigating children’s self-concepts from a cross-cultural perspective. Previous studies across collectivistic and individualistic cultures showed that multidimensional self-concept constructs could be studied cross-culturally thanks to culturally adjusted scales (Chen et al., 2020). The results of this study showed that the scores obtained from the social self-concept dimension did not differ between the children attending preschool education in two different countries. However, the scores taken from the ability-based and physical self-concept dimensions differed significantly in favor of the children attending preschool education in Turkey. The results found to be in favor of children in Turkey for the ability-based and physical self-concept dimensions were not predicted by the researchers’ hypothesis. Turkish families belonged to cultural contexts based on collectivistic cultural values while German families belonged to cultural contexts based on individualistic cultural values. In European countries, the relationship between parents and children tended to be more equalitarian (Garcia et al., 2020) and based on warmth and involvement (Gimenez-Serrano et al., 2022), so the parental pressure could be lower than in collectivistic cultural contexts (Chao, 2001). Therefore, in this study, the lack of difference between the social self-concepts of children attending preschool education in Turkey and Germany was a limitation of the study. The fact that there was no difference in the social self-concept can be interpreted based on the results of Miller (1984) cross-cultural study conducted on children in India and United States. In that study, Miller reported that there was no significant cultural difference in the way Indian and American children interpreted events, but this changed as the children get older. The cultural difference was not evident at very young ages, but the differences between cultures became more evident as age increased. The fact that there was no significant difference between the self-concept levels of the 36–72-month-old preschool children attending preschool education in two different countries can be explained by their being very young. This study was carried out with preschool children, and previous studies reported that there might be changes in self-concept during adulthood as age increased (Roberts et al., 2006; Bleidorn et al., 2009). In addition to this, self-concept studies adopting scales developed in the West can provide erroneous results for individuals living outside the West due to the possibility of prejudiced thoughts in favor of the West (Oyserman and Lee, 2008). In order to overcome this problem in this study, it was attempted to adapt the scale from Germany to Turkish culture, and a pilot study was carried out by obtaining the opinions of experts from Turkish culture as well as German experts. Although each study has a limitation, it is assumed that cultural prejudices are eliminated in this way. In addition to this, the results of this study were in parallel with the results of the study by Cai et al. (2007). Cai et al. (2007), although studies have persistently shown that self-esteem is lower in eastern culture, they stated that it was not true that countries in Eastern culture showed lower self-esteem than countries in Western culture. To prove this, they conducted two studies with Chinese individuals with a collectivist culture and with American individuals with an individualistic culture. The self-esteem of Chinese individuals was found to be lower than the self-esteem of American individuals in the first study. However, in the first study, cultural differences were ignored in the measurement tool measuring the self-esteem of individuals, and the scale was applied without adapting it to culture. In the second study, the scale was applied to the individuals in accordance with their own cultures by considering cultural differences. The result of the second study showed that Chinese individuals also felt positive about themselves like American individuals, but they did not reveal this much as required by their culture. In this regard, individuals in collectivist cultures cannot always be expected to have lower self-concepts than individuals in individualistic cultures. Even though they are in the collectivist cultures, especially preschool children are in a period that Piaget (1969) calls egocentrism. As a characteristic of this period, children prioritize themselves before the thoughts of those around them. Even though children in the egocentric period live in a collectivist culture, they will not show the behavior of caring much about the thoughts and social norms of others, which is the characteristic of this type of culture, as much as adults. In addition to all these, the strong relational ties in the collectivist Turkish culture and the fact that individuals prioritize the well-being of other individuals in the same environment (Kagitcibasi, 2010) may facilitate others’ involvement in the child’s own affairs in ability-based areas and areas where the child is physically challenged. On the other hand, in Germany, where the individualistic culture is dominant, parents’ belief in the importance of each individual acting and experiencing success and failure independently (Hofstede, 2001) may cause less intervention in children’s difficulties in ability-based and physical areas. In terms of an individualistic culture, parents in Germany consider it important for children to handle their affairs independently. However, sometimes children may not be equipped with the necessary skill to cope with every difficulty on their own without assistance due to their developmental characteristics. On the other hand, external intervention whenever children face various difficulties may cause the child to become dependent on help or develop a self-concept that does not reflect reality. However, providing support to children when they have difficulties in some physical activities due to their developmental process with solutions specific to the situation may help them experience the sense of failure less. This will support the positive development of children’s self-concept.

Male and female children are approached by adults with different expectations according to their gender within the framework of each culture’s own values, and this plays an important role in developing children’s self-concepts in accordance with these expectations (Kagitcibasi, 2010). Society’s expectations that differ according to child’s gender lead to differentiating levels of positivity between children of different genders in the dimensions of self-concept (Aoyagi et al., 2018). Studies showed that males had a higher level of positivity than females in the self-concept, which was defined as physical and academic or ability-based (Del Rio et al., 2019; Niepel et al., 2019). In this regard, the hypothesis of the study predicted that males in both countries would get higher scores than females from the ability-based and physical self-concept dimensions. Furthermore, the social self-concept was expected to be the same in females and males. The results of this study showed that this hypothesis was partially acceptable. Considering the ability-based self-concept dimension, it was determined that the scores of the 36–72-month-old females and males in Turkey showed significant differences by gender. This significant difference was in favor of the males. In the social self-concept and physical self-concept dimensions, the scores obtained by the children in Turkey did not differ significantly by gender. Previous studies also showed that males were more optimistic than females in the ability-based self-concept dimension (Ehm et al., 2011; Del Rio et al., 2019; Niepel et al., 2019). In Turkish culture, males and females are raised in accordance with the gender roles assigned to specific genders. Females are mostly supported in social competencies, while males are supported more in ability-based competencies (Cimen, 2000). In a study conducted in Pakistan with collectivist culture, it was determined that children were influenced by gender roles, had a collectivist self-structure, and their self-concept varied according to gender roles (Ahmed and Zaman, 2019). Preschool children can internalize gender stereotypes (Ambady et al., 2001). For example, females may adopt the belief that males are more successful in mathematics due to cultural stereotypes and develop a negative self-concept toward mathematics just because of being a girl (Baron et al., 2013). The fact that the males’ scores in the ability-based self-concept dimension were higher than those of the females can be interpreted within this framework. The scores of 36–72-month-old children attending preschool education in Germany did not differ significantly by gender in the dimensions of ability-based self-concept, social self-concept, and physical self-concept. This result suggested the possibility that gender stereotypes were not as common in Germany as in Turkish culture. Arens and Hasselhorn (2013) also reported that the self-concepts of the 3rd, 4th, 5th, and 6th-grade German students did not change according to gender. On the contrary, another study conducted in Germany showed that the academic self-concept of primary school children varied by gender. Accordingly, it was determined that males had a better academic self-concept than females although males and females obtained similar scores in the academic field (Heyder et al., 2019). On the other hand, various studies on self-concept in preschool children supported the conclusion that self-concept did not differ according to gender (Onder, 1997; Turasli, 2006; Zincirkiran, 2008). In this study, the self-concept of the children was compared by gender in their own countries and, then, they were subjected to a gender-based comparison between the two countries. When children were compared according to countries based on gender, the females attending preschool education in Turkey obtained higher scores than the females attending preschool education in Germany in the physical self-concept dimension. In the same dimension, it was also determined that the males in Turkey obtained higher scores than the males in Germany. There was no significant difference in the ability-based self-concept and the social self-concept between children in the two countries based on gender. Similarly, it was determined in another study that the physical self-concept scores of children in the collectivist Amazigh culture were higher than the scores of children in the individualistic European culture (Herrera et al., 2020).

### Strengths and Limitations and Future Research

Studies investigating the concept of the self across cultures often focus on adults. This may be due to the difficulty of measuring self-concept in the preschool period. It is considered that this study will make unique contributions to the literature as it was conducted by measuring the self-concept both verbally and concretely through one-to-one administration of the scales to children and by allowing the children to define themselves. In addition to this, special care was taken to adapt the Self-concept Questionnaire for Children to Turkish culture before it was implemented in Turkey.

One of the limitations of this study was that there was not an equal number of children participating in both countries. It is recommended to carry out a similar study with a greater number of participants. However, it was not possible to reach more children in this cross-cultural study although it was carried out with a one-to-one scale applied to 36–72-month-old children from many preschool education institutions. The related scale was implemented with meticulous effort and diversity was ensured by choosing the educational institutions from different socioeconomic levels and environments. This was one of the strengths of this study.

In this study, the self-concept levels of children in two different countries with collectivist and individual cultures in the ability-based, social, and physical dimensions were observed. However, it is not known whether children’s self-concepts reflect the ideal selves they want to have or the selves they already have. For this reason, studies on self-concept should be discussed with a holistic perspective and evaluated in light of information obtained from children, parents, and teachers.

First and foremost, self-concept in children is shaped as a result of communication with their family, then with their teachers and friends. Therefore, it is important to eliminate the possibility of parents and preschool teachers negatively affecting children’s self-concept or creating an imaginary self-concept by using excessive expressions of appreciation for children. In-service training can be provided to preschool teachers to create realistic and positive self-concepts in children so that they can support and develop children’s self-concepts in a healthy way instead of negatively affecting them. Families can be informed of the development and importance of self-concept through family participation studies and training to be provided by teachers.

To have a positive effect on the self-concept of preschool children, elective courses can be opened at universities, and teacher candidates can be trained on the importance of self-concept, the need for supporting it, and how to support it. In this way, teacher candidates will be able to recognize the children who have a lower self-concept level or are at risk more easily and support their self-concept more consciously when they start their profession.

A longitudinal study can be conducted to investigate the effectiveness of preschool education on self-concept development. In this longitudinal study, an experimental and control group can be formed, and the Self-concept Questionnaire for Children can be administered to children in both groups at the beginning and end of the study with a pre-test – post-test application. The preschool education curriculum can be evaluated in the light of the results to be obtained. Thus, the strengths and weaknesses of the curriculum to support positive self-concept development in preschool children can be revealed in more detail.

## Conclusion

In summary, this study investigated the self-concept levels of the 36–72-month-old children in preschool education in Turkey and Germany and determined whether there was a significant difference between children’s self-concepts or not. Accordingly, it was determined that there was a significant difference in favor of Turkish children in the dimensions of ability-based and physical self-concept. It was determined that there was no significant difference in the social self-concept between the countries. Considering the results in terms of gender, it was determined that the social self-concept and physical self-concept scores of the females and males in Turkey were not significantly different and ability-based self-concept differed in favor of the males. It was determined that children in Germany did not differ significantly according to gender in the dimensions of ability-based, social, and physical self-concept. When children were compared according to countries based on gender, it was determined that there was no significant difference in the ability-based self-concept and the social self-concept between the children in the two countries. However, it was also determined that females attending preschool education in Turkey had higher physical self-concept dimension scores than the females attending preschool education in Germany. In the same dimension, males in Turkey were also found to have higher scores than the males in Germany.

It was concluded that Turkish children’s ability-based and physical self-concepts were higher although German children’s self-concepts were expected to be higher. It was considered that this was because social values were more important than individuals in collectivist societies. Respect and obedience to adults and the elderly (primarily family members) are also important for social values in collectivist cultures. Moreover, adults with collectivist cultures do not highlight the positive aspects of child’s behavior to ensure that they learn modesty. Therefore, it was surprising that the Turkish children’s self-concept scores were higher than those of German children. It was also significant that this difference appeared specifically in the ability-based dimension as a result of the social values attaching special importance to males in Turkish society.

## Data Availability Statement

The original contributions presented in the study are included in the article/Supplementary Material, further inquiries can be directed to the corresponding author.

## Ethics Statement

The studies involving human participants were reviewed and approved by the Hacettepe University (Ethics approval number: 35853172/433-816). Written informed consent to participate in this study was provided by the participants’ legal guardian/next of kin.

## Author Contributions

BC: conceptualization, investigation, original draft preparation, data collection, data analysis, methodology, and writing. AÇ: conceptualization, supervision, reviewing, and editing. Both authors contributed to the article and approved the submitted version.

## Conflict of Interest

The authors declare that the research was conducted in the absence of any commercial or financial relationships that could be construed as a potential conflict of interest.

## Publisher’s Note

All claims expressed in this article are solely those of the authors and do not necessarily represent those of their affiliated organizations, or those of the publisher, the editors and the reviewers. Any product that may be evaluated in this article, or claim that may be made by its manufacturer, is not guaranteed or endorsed by the publisher.
